# “Micro-managing” immune activation and protein turnover: microglial lysosomes in the context of health and disease

**DOI:** 10.1038/s44400-026-00086-8

**Published:** 2026-04-29

**Authors:** Elizabeth A. Somodji, Swetha Gowrishankar

**Affiliations:** 1 Department of Anatomy and Cell Biology, College of Medicine, University of Illinois Chicago, Chicago, IL USA; 2 Department of Biological Sciences, College of Liberal Arts and Sciences, University of Illinois Chicago, Chicago, IL USA

**Keywords:** Cell biology, Immunology, Neuroscience

## Abstract

Lysosomes, central to protein and organelle homeostasis in all cells, have more recently been recognized as critical to other cellular processes, including nutrient sensing, cell metabolism, immune response, and programmed cell death. Tied into these varied functions, their composition as well as location within cells, lysosomes are now recognized to be made of heterogeneous subpopulations. Mechanisms that build and help maintain lysosome heterogeneity, its role in cell physiology, and links to pathologies are only now being worked out. Lysosome dysfunction has been associated with the pathogenesis of several neurodegenerative diseases. Neurons, which are particularly sensitive to lysosome dysfunction, are perhaps where lysosome biogenesis and transport, as well as its heterogeneity, are best studied in the nervous system. However, there is a growing interest in understanding lysosomal biogenesis and trafficking in other cell types of the nervous system, including microglia. In this review, we focus on key studies that have shed insight into microglial lysosome biology: its regulation, composition, and function, and how these properties are linked to immune activation, aging, and certain disease pathologies.

## Introduction

Microglia are resident immune cells in the central nervous system and thus a critical and often first line of defense for the brain. They are involved in eliminating microorganisms, dead cells, aggregated proteins and synaptic pruning^1–4^. Thus, microglia are involved in both development and maintenance of a healthy central nervous system, including maintaining normal neural circuitry, and in aiding repair, post-injury^4,5^. Several key physiological features help microglia to execute these myriad functions; these include migration, metabolic signaling, phagocytosis and degradation^6^. A robust endo-lysosomal system is quite critical to microglia to carry out these clearance functions, and there are now numerous links between aberrant lysosomal pathways in microglia and deficits/perturbations to phagocytic, neuroinflammatory pathways in neurodevelopmental and neurodegenerative disease^7,8^. While much of the research into proteostasis pathways in the context of neurological diseases is focused on neurons^9–11^, there is growing interest in understanding and potentially harnessing microglial lysosomes for therapeutic intervention^8^. Interestingly, studies on neurological disease have in turn shed insight into the makeup and regulation of microglial lysosomes^7,12,13^. In parallel, with the expanding functional repertoire of lysosomes^14,15^, there is a growing appreciation for heterogeneity within lysosomes^16,17^ pertaining to not just their function, but also their composition and positioning within cells.

In this review, we discuss recent insights on regulation, biogenesis, and functioning of microglial lysosomes, in the context of immune activation, aging, and disease pathology, and evidence for lysosomal heterogeneity in microglia, as well as what the key future research directions in this field are likely to be.

## Network-level regulation of microglial lysosomes

Given that microglia are central to immune clearance of pathogens and cellular debris in the brain, processes which require robust lysosome function, there is considerable interest in understanding mechanisms that regulate lysosomal function in these cells and potentially enhancing lysosomal function in them in the context of neurodegenerative diseases^8^. The transcription factor EB (TFEB) and other members of the MITF family, since their discovery^18^, have been shown to activate diverse lysosomal processes, including lysosome biogenesis, lysosomal exocytosis, and autophagy^14^. Briefly, this family of transcription factors moves into the nucleus to activate the “CLEAR”/“Coordinated Lysosomes Expression and Regulation” network, inducing autophagy and lysosomal genes, to promote catabolic turnover of proteins and organelles under conditions of stress such as starvation or lysosome dysfunction^14,18,19^. However, relatively little is known about the role of these transcription factors in microglia^20,21^, given how much these cells depend on lysosomal pathways to execute their key functions. An interesting study utilized different mutant zebrafish to activate and suppress TFEB pathways in combination with live imaging, to demonstrate that a Rag A GTPase- and FLCN-dependent lysosomal regulatory circuit suppressing TFEB is necessary for proper migration of glia to the brain and their immune response^22^. While *raga* and *flcn* deficient mutants exhibited expanded lysosomal compartments in their glia^22,23^, they also had an excess of apoptotic corpses in the brain, suggestive of reduced glial clearance. These phenotypes were, in turn, rescued on deletion of *tfeb* and *tfe3* in *raga* mutant, while *tfeb* overexpression phenocopied the *raga* mutants. Lastly, they demonstrated that *tfeb* and *tfe3* were dispensable for basal expression of lysosomal genes in glia but were necessary for transcription of these genes in response to stress induced by injected Zymosan A^22^.

Consistent with a role for microglial TFEB-mediated target activation under stress conditions, TFEB-mediated up-regulation of V-ATPase activity has been shown to play a role in the immune response of microglia in a Tau mouse model (called PS19)^13^. Here, unlike many studies that examine the effect of exogenous TFEB expression, the authors relied on mutating the TFEB-binding promoter region (CLEAR sequence) of the endogenous mouse *atp6v1h* gene (named CL mutant), which encodes the corresponding V-ATPase subunit, to specifically perturb the TFEB-VATPase axis. The authors focused on this axis, based on the observation that firstly, bulk RNA sequencing data from the hippocampus of mice with Tau aggregation led to upregulation of lysosomal genes, and subsequent snRNA sequencing that pointed to a microglial cell cluster that had upregulation of genes encoding several of the *v-ATPase* subunits. Assays in cultured microglia from the CLEAR mutant and WT mice versus a KO of the subunit (V-KO) revealed that the CLEAR mutant glia had defective lysosomal acidification and proteolytic activity, unlike the KO, suggesting that this TFEB-V-ATPase axis, rather than steady state lysosomal gene expression, was responsible for modulating V-ATPase activity and lysosomal function in these cells. Lastly, crossing the CL mutant mice with the PS19 mice, and examining CD68 and Iba1 expression in them revealed that glial response, as measured by both these markers, was far less in the CL mutant background as compared to even the V-KO; Tau mice. Thus, in a second in vivo context, the TFEB-mediated program (TFEB-V-ATPase axis in the latter), is critical for the glia to mount an immune response under conditions of stress and/or protein aggregation^13^.

Another example of network-level regulation of glial lysosome function comes from a recent study on the Parkinson’s Disease (PD)-linked kinase, LRRK2^24^. The study demonstrated that LRRK2 acted through regulating the abundance and nuclear localization of MITF-TFE transcription factors to suppress lysosomal protein expression and degradative activity in macrophages and microglia. LRKK2 KO or its pharmacological inhibition in iPSC-derived macrophages increased expression of multiple cathepsins in these cells, both at a transcript and protein level. This was dependent of MITF-TFE family of TFs as TFE3 was localized to the nucleus in LRRK2 KO macrophages, and depletion of these TFs negated the lysosomal changes arising from LRRK2 loss. Interestingly, this nuclear localization of MITF/TFE factors, was not observed in LRRK2 KO iNeurons, suggesting that this is microglia-specific regulation. Lastly, the PD-linked hyperactive LRRK2 (G2019S) reduced lysosomal degradative activity in iPSC-derived microglia, suggesting that this reduced lysosomal degradative activity within glia due to LRRK2 hyperactivity could increase PD-risk^24^.

In summary, while stressors appear to induce TFEB-mediated lysosomal changes within microglia, mechanisms that suppress TFE-mediated pathways appear to operate to maintain homeostatic conditions in these cells.

## Microglial lysosomes in context of immune response

**Fig. 1:**
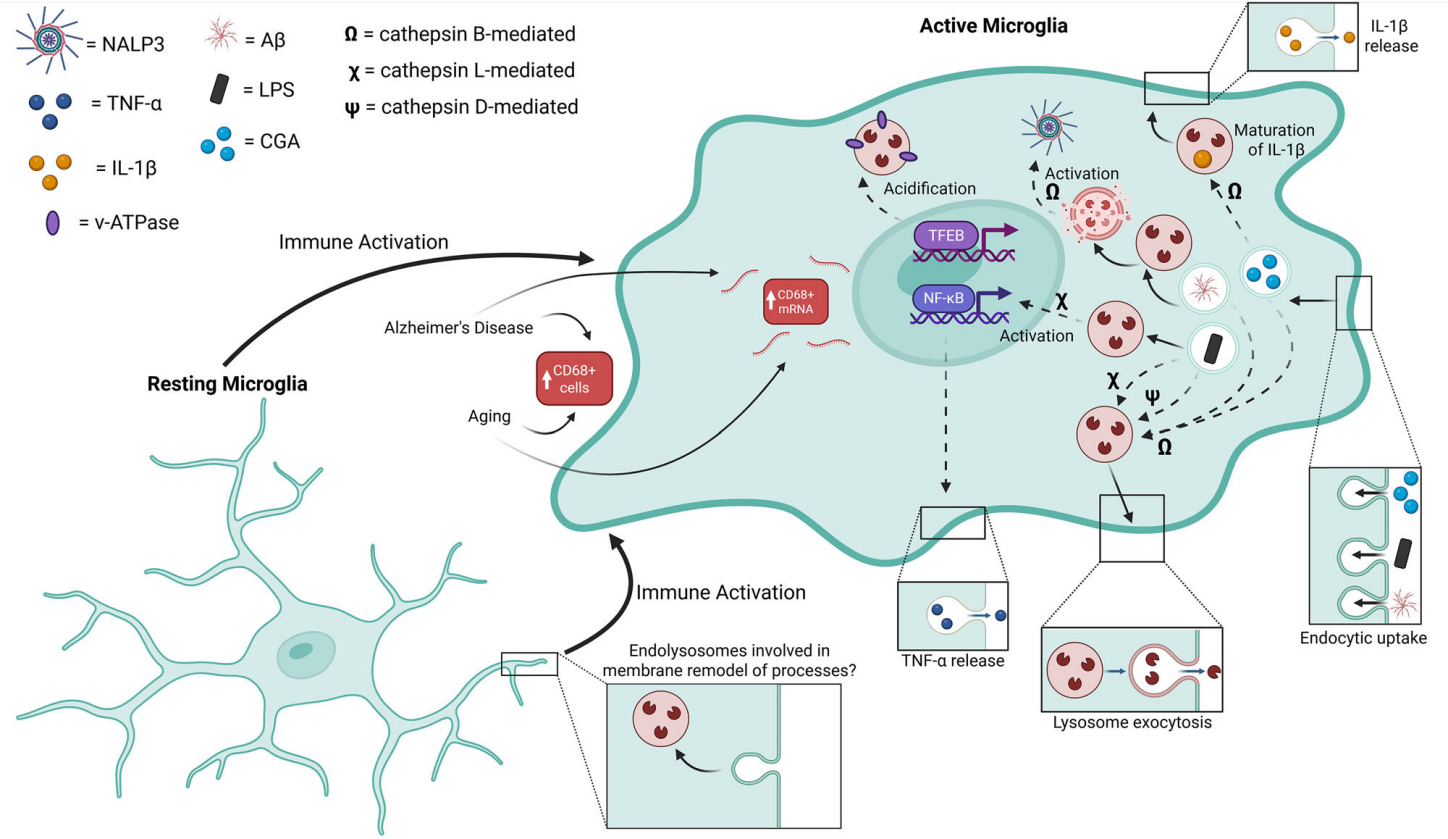
Microglial lysosome regulation during immune activation. Schematic of lysosomal and downstream immune pathways in microglia that are initiated under stress [e.g., Alzheimer’s Disease (AD), aging, CGA or LPS uptake]. The transition from resting microglia to activated glia involves morphological changes where glia move from ramified to more ameboid morphology with reduced processes. The role of endo-lysosomes in effecting this membrane remodeling remains to be determined. In AD and aging, there is increased CD68 mRNA in glia and an increase in CD68+ glial cells. TFEB-mediated lysosome acidification is observed in microglia under stress conditions. Upon uptake of immune-stimulating proteins (e.g., CGA, LPS, or Aβ), activated microglia lysosomal pathways play a critical role in mobilizing immune responses. Key cathepsins that mediate these immune responses include **a** cathepsin B (Ω): aiding in the maturation of IL-1β in CGA-stimulated glia and **b** cathepsin L (χ) mediating the activation of transcription factor NF-κB and downstream expression of TNF-α in LPS-stimulated cells. The uptake of Aβ can also induce lysosome damage, where the release of cathepsin B can mediate the activation of the NALP3 inflammasome. Lysosomes in activated microglia also undergo exocytosis, which is likely how CGA and Aβ-stimulated glia release cathepsin B into the extracellular matrix, and likely how LPS-stimulated cells release cathepsin L and cathepsin D (ψ). Dashed arrows indicate there may be more steps/signaling downstream, leading to the depicted effect.

Lysosomes, in addition to being central players in proteostasis within cells, also carry out myriad other functions, including processing and secretion of inflammatory signals, being the destination of phagocytosed pathogens or foreign material^14,25^. The link between lysosomes and immune response is corroborated by both network-level changes to lysosomal pathways with immune activation under stress conditions as detailed above^13,22^, as well as with evidence for change in specific lysosomal proteins and/or properties upon immune activation^2,26^ (Fig. 1). In further support of a strong interplay between immune response and lysosomal upregulation, perturbation to key immune-related proteins also cause lysosomal changes^26^. This includes changes associated with perturbation to TREM2, a transmembrane receptor that is part of the immunoglobulin superfamily^27,28^, is highly expressed in macrophages and microglia, and binds to a variety of ligands including DNA, lipoproteins (such as APOE), phospholipids, and Aβ, to activate downstream signaling cascades^27,28^. Induced microglia (iMGs) with TREM2 p.Q33X mutation or TREM2 KO, exhibited significant downregulation of several endolysosomal genes including RAB7A, LAMP1, LAMP2, CD68, CD63 and CTSS^26^. Additionally, fluorescence assays in these TREM2 mutant glia that measured lysosomal acidification and proteolytic activity, indicated decreased lysosomal acidification and degradation in microglia lacking TREM2 function.

Given neuroinflammation that is associated with AD^29^, and links between immune activation and lysosomes, there is a growing interest in developing therapeutics targeting microglia-specific lysosomal pathways. A study where aged mice were subjected to a five-month oral treatment of ketotifen, an approved H1-antihistamine, led to re-acidification of lysosomes, reduced cytokine release in in vitro assays, and restored microglial morphology to a more homeostatic, ramified state^30^. Of broader relevance, a recent study determined that the Alzheimer’s therapeutic, Lecanemab, elicited its amyloid clearance through activating clearance pathways in microglia^31^. Here, the authors used single-cell RNA seq and spatial transcriptomics to show that lecanemab induces targeted phagocytic, lysosomal degradation pathways in microglia in their human microglia xenotransplantation AD mouse model^31^.

Enhancement or upregulation of lysosomal genes because of immune activation suggests that components of the organelle likely contribute to the downstream immune response. An important class of lysosomal proteins implicated in this are cathepsins. Cathepsins are a major class of proteases within lysosomes^14,32,33^ that aid in the organelle’s degradative activity^32^. Modulation of expression and/or activity of cathepsins in neurons and microglia have been linked to many neurological diseases^34–37^. Given their role in protein breakdown, dysfunction or lack of some of the cathepsins has been associated with neurodegenerative diseases that are characterized by protein aggregates^14,34–37^. Some studies suggest that certain cathepsins, such as Cathepsin B (Cat B), are anti-amyloidogenic, aiding in cleavage of Aβ42, and thus neuroprotective^35,38,39^. However, in addition to their role in protein breakdown, microglial cathepsins, particularly when secreted^40^, have been linked to neuroinflammation (Fig. 1).

A critical role for Cat B in glial inflammatory response is, in fact, supported by several studies^38,41,42^. In vitro studies of Chromogranin A (CGA)-treated primary microglia revealed that Cat B transcript, protein levels increased upon the treatment, and that Cat B was necessary for the maturation of IL-1β^41^. Immunostaining experiments showed a strong overlap of Cat B, caspase-1 and the cleaved IL-1β, with large acidic lysosomes, suggesting that the caspase 1 activation occurred within acidic lysosomes in CGA-treated cells (Fig. 1). In addition to the inhibitor-based studies, microglia within hippocampus of Cat B-deficient mice exhibited reduced immunoreactivity for the mature IL-1β, supporting the requirement of Cat B for cleavage and maturation of IL-1β^41^. A study where exogenous Aβ42 was injected into the hippocampus of either wild-type or Cat B-deficient mice suggested that Cat B is involved in the inflammation, cell activation, migration, and phagocytosis-linked transcriptional changes induced in these cells following Aβ42 exposure. Indeed, functional assays that measured these properties revealed that the lack of Cat B reduced microglial migration in an in vitro scratch assay and Aβ42-induced inflammatory response^38^. Cat B release from lysosomes has also been linked to age-dependent ROS generation and inflammation^42^. Here, higher levels of ROS were detected in the hippocampus of aged wildtype mice when compared to Cat B-deficient mice^42^. Some studies support the release of Cat B outside of the microglia via secretion, as observed in CGA-stimulated primary rat glia^40^ and Aβ42-treated murine BV2 microglia cells^43^. In both these cases, the glial Cat B release was linked to neuronal cell death^40,43^. Interestingly, one study did identify a Cat B isoform unique to microglia^44^. They found that BV-2 cells have both a 32 kDa cathepsin B (isolated at a pI of ~5.5–5.2) and 34 kDa cathepsin B (isolated at a pI of ~5.1–4.5), unlike the single form they observed in samples of liver cells and whole brain lysates^44^. The difference in pI values observed in the Cat B isoenzymes might suggest that the isoforms could function best at different pH values, which would correlate with the acidic lysosomal lumen versus the extracellular environment. Indeed, they found that while one form was stable at pH 7 and was degraded at the lower pH of 5.5, the opposite was true for the second isoform^44^. Cathepsin L (Cat L) has also been linked to inflammation, with LPS-stimulated BV-2 cells exhibiting increased Cat L, caspase-8 cleavage, leading to increased activation of NF-κB and downstream expression of proinflammatory TNF-α^45^. As with Cat B, LPS-stimulated primary rat microglia have also been shown to release Cat L into the extracellular mileu^46^. LPS-stimulated and IFN-γ-treated BV-2 cells also release Cat D into the extracellular matrix^47^. Interestingly, in LPS-primed MG6 microglia, a specific form of IL-1β (20 kDa form) is produced by Cat D cleavage, when both are released separately in the extracellular matrix, the former from the cytosol, while the latter through calcium-dependent lysosomal exocytosis^48^. Immunostaining confirmed that the Cat D within lysosomes doesn’t contact/colocalize with the IL-1 β within the microglia. The cleavage was shown to be Cat D-dependent, as Pepstatin A treatment inhibited, while treatment with Cat B -inhibitors had no effect. Taken together, there is strong evidence to support a critical role for multiple lysosomal cathepsins in pro-inflammatory response upon microglial activation (Fig. 1).

Another major microglial lysosomal protein that has been linked to both inflammation and degradation is the Cluster of Differentiation 68 (CD68) protein, which we discuss in depth in the next section.

## CD68: convergence of phagocytic, inflammation, and lysosomal clearance pathways in microglia

CD68 (Cluster of Differentiation 68) is a type I transmembrane glycoprotein belonging to the LAMP family of glycoproteins (e.g., LAMP1)^49–52^, that is highly expressed in cells from the mononuclear phagocyte lineage that includes microglia and macrophage^50,52^. Much of the information on CD68 function has come from studies involving macrophages^53–55^. It is considered a scavenger receptor that binds to oxidized low-density lipoprotein (oxLDL)^56,57^ and purported to be expressed on the cell surface in small amounts due to rapid turnover^54,56^. However, CD68 is also known to be expressed on endosomes/lysosomes in both macrophages^52,58^ and microglia^52,59^, and is often used as a marker for phagocytic/active microglia^59–63^. Studies in mice have revealed that CD68 levels in microglia increased with age, in a region-specific manner, that appeared to correlate with oxidative stress, and was attenuated upon caloric restriction^64^. The link to oxidative stress is further supported by in vitro experiments in BV-2 cells, where CD68 mRNA and protein levels increased when exposed to oxLDL^64^. Interestingly, CD68 mRNA expression and number of CD68+ glia increase in AD^62,65^, which is also characterized by oxidative stress and inflammation, further strengthening an association between CD68 and these processes (Fig. 1). Together, this would suggest that alterations of CD68 are not just linked to pathology but also in the physiological context of aging.

However, while CD68 labeling has been used as a readout of phagocytic activity^62^ the precise role of CD68 in microglial phagocytosis is still not fully clear^56^. One study demonstrated that glycosylation of CD68 expressed in macrophages occurs specifically during phagocytosis, as there was increased glycan labelling with phagocytic material (e.g, zymosan) rather than endocytic material (e.g., mannosylated BSA)^55^. While CD68 can bind to oxLDL, it is unclear if and how microglial CD68 is specifically involved in the uptake of oxLDL^53,54^.

Iba-1 is most used as a microglial marker; it is sometimes co-stained with CD68, the latter labeling lysosomes within these cells. A study that examined both these markers along with MHC-II in tissue from Control, NAWM (Normal Aged White matter), and DSCL (Deep subcortical Lesion) found that there was no major difference in MHC-II levels between them, but there was a step-wise increase in CD68 levels from Control to DSCL, and Iba1 levels were higher. A striking feature of DSCL, which is observed in 60% of individuals over 60 years of age and is linked to depression and cognitive decline^66^, was the presence of a population of iba1-/CD68+ microglia^67^. This study reiterates the need for possibly co-staining for multiple markers when examining microglia, especially in the context of human disease pathology, and that CD68 is a critical marker for detecting changes in microglia. In summary, changes in CD68 (increased immunoreactivity) have been documented in the context of AD, ageing (Fig. 1), which are both associated in turn with oxidative damage, and while its expression has been linked to inflammation and lysosomal clearance, the precise role of CD68 in microglial lysosome function has not been elucidated.

## Differential effects of disease mutations and perturbation on microglial and neuronal lysosomes

The dual key roles for microglial lysosomes in mounting an immune response and proteostasis versus neuronal lysosomes being more primarily involved in protein turnover could explain why perturbations to several lysosomal proteins have different effects on microglia as compared to neurons. For instance, while loss of PGRN, a glycoprotein that is highly expressed in glia and select neurons in the CNS^68,69^ causes lysosomal maturation defects in microglia, its loss causes a concomitant upregulation of several lysosomal proteases in other cell types in the brain, suggestive of different cell-type-specific functional consequences on lysosomal homeostasis^70^. PGRN, while reported to play roles in neurotrophic signaling when secreted, has more recently been linked to lysosomal degradative activity through its regulation of multiple cathepsins as well as glucocerebrocidase^68^. Of relevance to Alzheimer’s Disease, increasing PGRN expression in primary wild-type microglia increased endocytosis of Aβ^71^. PGRN deficiency in microglia compromised autophagy and caused a three-fold increase on plaque load in a mouse model of AD^72^. Loss-of-function variants of Sortilin Related Receptor protein or SORLA are causally linked to AD^73,74^. This VPS10P receptor, which is involved in intracellular protein sorting through its cycling between the TGN, endo-lysosomes, and the cell surface^75^, has primarily been characterized in neurons. Interestingly, while a study on SORL1 KO iNeurons revealed enlarged early endosomes^76^, its loss from iMGs seemed to cause enlarged late endosomes and lysosomes^73^. A recent study that made use of SORLA KO human iMGs showed through cellular and biochemical assays that loss of the receptor in microglia led to reduced lysosomal enzyme activity without changes to their expression levels^73^. Likely due to this, the cells also exhibited decreased cargo degradation. In vitro assays that examined uptake of fibrillar Aβ1-42 and synaptosomes revealed that loss of SORLA led to increased uptake and accumulation of these cargoes but not of oligomeric Aβ42, suggesting substrate specificity for SORLA in microglia. Interestingly, in response to calcimycin-induced acute stress, the KO iMGs released lesser amounts of ILs (IL-1β and IL-6), suggesting a stunting of the inflammatory response that could possibly tie into the lysosomal defects. A recent study demonstrated that both the hexanucleotide expansion and KO of C9Orf72 KO in iMGs leads to transcriptional changes in endolysosomal pathways^77^. The mutations in the gene encoding C9ORF72 protein are linked to ALS and FTD^78^, and the protein itself localizes to lysosomes and regulates endo-lysosomal and autophagy pathways^79^.KO iMGs exhibit clustering of Cathepsin D-positive lysosomes compared to their control. However, the physiological consequence of this clustering has not been worked out. In vitro assays showed impaired breakdown of phagocytic material in the KO microglia, suggestive of lysosomal dysfunction.

Mutations in the gene encoding PS1, a subunit of the γ-secretase complex that is involved in processing APP, have been causally linked to AD. PS1 perturbations have been extensively studied in neurons^80^. Interestingly, PS1 is highly expressed in microglia within the mouse brain cortex^81^. While a phosphor-deficient PS1 caused defects in autophagosome-lysosome fusion in neurons^82^, studies in a knock-in mouse model of this same phospho-deficient PS1 (S367A) showed that altered PS1 caused altered microglial morphology, including reduction in number and length of ramifications, as well as reduced ability to surveil their environment^80^. When cultured PS1 mutant microglial cells were challenged with oligomeric Aβ42, they exhibited no defects in uptake of the oligomers but did have impaired ability to degrade them. Consistent with this, in a 5xFAD model, with PS1 KI, the microglia exhibited higher Aβ42 levels, and there was increased plaque burden in several brain regions of these animals. Of note, the number of microglia enveloping each plaque was comparable to 5xFAD animals, suggesting the PS1 mutation didn’t alter their recruitment to plaques, but did alter Aβ42 metabolism. Alterations to an adaptor protein complex, AP-4, that has been shown to regulate lysosomal function and composition in neurons^83^, caused a similar exacerbation of amyloid plaque burden, but was accompanied by increased recruitment of microglia to these plaques^84^. The effect of loss of AP-4 complex function on microglial lysosome biology and its links to immune activation remains to be determined. As detailed earlier, deficiencies in cathepsins have different consequences in microglia as compared to neuronal lysosomes.

## Lysosomal stress and damage in microglia

Given their role in degradation and turnover of material that can include toxic protein aggregates, lysosomes are particularly vulnerable to membrane permeabilization or rupture^85,86^. This damage can occur due to a variety of agents and cellular conditions, including lipid changes, accumulation of lipid metabolites, phagocytic uptake of sphingomyelin-rich material, ageing, toxic aggregates of species including Tau, α-synuclein, β-amyloid fibrils. The resulting lysosomal membrane permeabilization (LMP) leads to the release of lysosomal proteases into the cytoplasm, which in turn triggers cell death pathways^86^. Cells have developed countermeasures to protect from the consequences of LMP, which include restoring the lysosomal membrane permeability barrier/lysosome repair, consumption of damaged lysosomes through lysophagy, and, lastly, generation of new lysosomes. In recent years, studies in several cell types have shed light on the molecular mechanisms underlying the sensing of lysosomal damage and the subsequent response, and this has been reviewed extensively^85,87,88^. Interestingly, a recent study that examined mobilization of repair mechanisms upon LLOMe-induced lysosomal damage in primary neuron-astrocyte co-cultures determined that astrocytes engaged a broader repertoire of repair pathways than neurons^89^. This difference in pathways that were activated could not be explained by just differences in protein expression of components of the repair machinery in the two cell types. These results raise an interesting possibility of differential lysosome resilience in the distinct cell types of the CNS, which could contribute to selective vulnerability with aging and neurodegenerative diseases. In fact, a study that carried out proteomic analysis of transdifferentiated neurons obtained from aged and AD fibroblasts identified lysosome damage and defects in ESCRT-dependent lysosome repair machinery as an early pathogenic feature of these neurons^90^. Given their phagocytic activity and involvement in inflammatory response, microglial lysosomes are potentially more likely to encounter lysosome-damaging agents and may also have more robust repair or damage-sensing mechanisms. Indeed, evidence from studies in the AD and immune activation context suggests that LMP could be a feature of microglial response to stress. In vitro experiments in BMD-microglia linked lysosome-damage-induced Cathepsin B release from lysosomes to NALP3 inflammasome activation^91^. Here, labeled Aβ uptake in cultured glia caused the Cathepsin B, initially seen in punctate LAMP1-positive vesicles, to become more diffuse and primarily localize outside swollen LAMP1- vesicles, suggesting it was released from damaged lysosomes^91^. In support of a robust repair /counteractive pathway in microglia, levels of Cystatin B, a cytoplasmic neuroprotective factor, and inhibitor of Cathepsin B are found to be elevated in activated microglia in the spinal cord of an ALS mouse model^92^. Another aggregation-prone protein, a-synuclein in its fibrillar form (f-AS), was demonstrated to induce lysosomal damage in both primary murine microglia and BV2 glial cell line^93^. Through live imaging and CLEM across time, the authors showed that the f-AS induced lysosomal damage rather than f-AS itself, induced autophagy in these cells, in a manner dependent on autophagy proteins, Optineurin and TBK1. This autophagy induction, a means to clear out damaged lysosomes, was critical to glial cell survival, as inhibiting autophagy through genetic or pharmacological means in f-AS-treated microglia led to cell death. Intriguingly, f-AS, while causing lysosomal damage within microglia, did not damage mitochondria, unlike what was previously reported in neurons. These results are also suggestive of differential sensitivity to damaging agents and /or different pathways getting triggered, in the distinct cell types. It will be interesting to determine how microglial lysosomes respond to damaging agents when directly compared to neurons and astrocytes utilizing co-cultures. Likewise, it will also be important to determine if microglial lysosome repair mechanisms are altered with age and in different disease states, as reported in neurons^90^.

## Lysosomal heterogeneity within and between microglial cells

Prior to delving into lysosomal heterogeneity within a single cell, it is important to recognize that microglia themselves exhibit heterogeneity in varying contexts^94–96^. Decades of work have shed light on the heterogeneity of microglia in terms of their morphology, electrophysiological properties, surface expression of a panel of immune molecules, and response to disease states^94,97–99^. However, the advent of novel techniques such as single-cell RNAseq has allowed the exploration of microglial heterogeneity at an unprecedented throughput and in an unbiased fashion^94,96^. Intriguingly, evidence for microglial heterogeneity based on lysosomal properties has come from a recent study that classified microglia into subtypes in the context of aging^100^. Here, the classification of microglia into subtypes correlated with lysosomal function and lysosomal protein expression. Burns et al. examined autofluorescence levels within microglia from healthy brains of mice and monkeys. Using FACS analysis, they found that microglia (CD45^*dim*^, CD11b^*+*^) fell into two main populations: the majority (70%), autofluorescence +(or AF+), and a smaller (30%) AF- population, with the AF signal arising from an intracellular organelle. Interestingly, examination of the signals from glia of aged animals revealed that there was a linear increase in AF signal within the AF+ population, along with expansion in volume of the intracellular compartments responsible for this signal. However, the proportion of AF- glial stayed mostly the same, and their AF signal did not increase with age. EM analysis revealed expansion of complex “lysosomal storage bodies” within AF+ glia, and IHC indicated increased LAMP1 and CD68 expression with age, only in these AF+ microglia. LC-MS of FACS-isolated AF+, AF- microglia revealed that 32 of 50 top differentially expressed proteins (DEPs) were related to endo-lysosomal trafficking. AF+ microglia exhibited upregulation of several Cathepsins (Cathepsins A, B, D, F, L, Z), glycosyl hydrolases, and proteins involved in lysosome motility and phagosome-lysosome fusion (TMEM55B and Arl8B) as well as the lysosome-linked Transcription factor, TFEB. Lastly, in the mouse brain, with advanced age, there was a specific and dramatic fall in % of AF+ microglia^100^. Thus, at the very least, subsets of microglia with differential lysosomal clearance capacities exist, and they change differently with age.

**Fig. 2:**
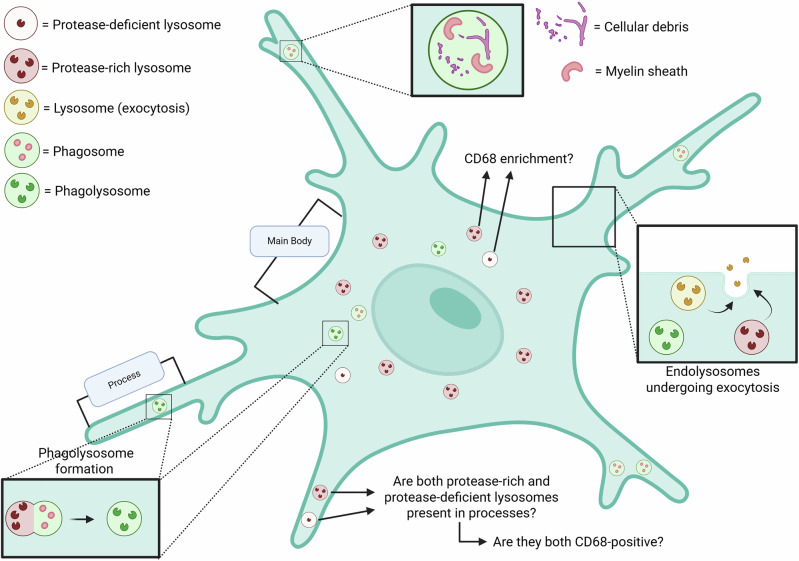
Properties of microglial endolysosomes. Schematic depicting known properties of microglial endolysosomes as well as critical characteristics and functions that remain to be determined. Both the main body and processes of microglia have endo-lysosomes, though questions remain as to whether they differ in any way in composition (including in protease content and CD68 enrichment), as observed in other polarized cells, including neurons. Phagosomes having inclusions, myelin sheath, and cell debris have been observed in both processes and the main body of microglia, and undergo fusion with endo-lysosomes. Future studies could help determine if there are unique sub-populations of lysosomes for specialized functions, such as those undergoing lysosome exocytosis and those involved in fusing with and consuming phagosomes, including how these functions correlate with protease content and CD68-positivity.

At the single cell level, lysosomes have come to be recognized as dynamic and functionally diverse organelles, composed of heterogeneous subpopulations within even individual cells, based on their composition, their position within the cell, as well as the physiological state of the cell^101^. While this is found to be the case even in non-polarized cultured cells, polarized cells with morphologically and functionally distinct sub-domains are likely to have different proteostasis demands and thus possibly more heterogeneous lysosomal populations. Indeed, the lysosomes within neuronal processes, namely, axonal and dendritic lysosomes, differ in their transport properties, response to neuronal activity, and cargo content^9,102^. The fact that microglia have numerous dynamic processes that are distinct from their main body, which are morphologically altered in response to the cell’s activation state as well as under certain pathological states, raises the possibility that they have different lysosomal populations (Fig. 2). These lysosomal populations could vary in transport dynamics as well as signaling components, from those within its main body, as is the case with neurons. Indeed, a study that examined the ultrastructure of organelles within the main body and processes of microglia, noted that there were more non-empty inclusions within phagocytic vesicles of processes versus the main body^103^. However, this study primarily focused on differences between phagosomes, lysosomes (in both main body and processes), between wild-type microglia and those in an AD mouse model, rather than within the glia itself. Future studies, which layer immunolocalization of key lysosomal proteins such as CD68 in this context, could shed new insight into how lysosomes between the processes and main body of microglia differ (Fig. 2). Furthermore, the established links between immune activation and alterations to microglial lysosomal properties including levels of certain lysosomal proteins and release of cathepsins, raises the possibility that distinct lysosomal populations may either already exist or form de novo in response to microglial activation. The identification of two different isoforms of Cathepsin B within microglial lysosomes^44^, that have differential stabilities at acidic pH, raises the question as to whether they are present in distinct lysosomal populations, with one being more prone to secretion. Increase in CD68, a lysosomal protein that has been linked to activation, phagocytosis, and aging, and near plaques, in AD. However, this has not been explored at the level of individual lysosomes. Advances in image acquisition and analysis would now allow us to examine how CD68-positive lysosomes change in these distinct contexts. This will also allow us to interrogate whether CD68 labels a subset of lysosomes (Fig. 2), such as those that could engage more with phagosomes. One way to determine this would be to analyze the level of colocalization of LAMP1 and CD68+ lysosomes in microglia. A study has examined this overlap; they partially overlapped, but this was carried out in macrophages, and the level of colocalization was not quantified, nor parsed out in terms of location within the cell^104^.

In summary, some of the outstanding questions and future directions for the field of microglial lysosomes include: (a) how do properties of lysosomes within glia at resting state differ from those in activated state?; (b) how do endo-lysosomes contribute to the rapid membrane remodeling that must occur within microglia during immune activation, to accomplish the dramatic morphological changes including reduction in glial processes (Fig. 2)?; (c) Does microglial activation trigger new lysosomes to form? (d) What is the precise role of CD68 on lysosomes in these contexts, and lastly (e) aspects relating to microglial lysosome heterogeneity, such as whether lysosomes in microglial processes are distinct from those in its main body; are there distinct subsets /types of lysosomes in microglia, with one more prone to secretion? Deciphering the answers to these questions will be important, from a basic cell biology perspective as well as in the context of understanding disease mechanisms.

## Data Availability

No datasets were generated or analyzed during the current study.
